# Attitudes and perceptions toward the use of artificial intelligence chatbots for peer review in medical journals: a large-scale, international cross-sectional survey

**DOI:** 10.1186/s41073-026-00256-5

**Published:** 2026-10-26

**Authors:** Jeremy Y. Ng, Daivat Bhavsar, Neha Dhanvanthry, Lex Bouter, Teresa Chan, Holger Cramer, Annette Flanagin, Alfonso Iorio, Cynthia Lokker, Hervé Maisonneuve, Ana Marušić, David Moher

**Affiliations:** 1 Institute of General Practice and Interprofessional Care, University Hospital Tübingen, Tübingen, Germany; 2 Robert Bosch Center for Integrative Medicine and Health, Bosch Health Campus, Stuttgart, Germany; 3 School of Public Health, Faculty of Health, University of Technology Sydney, Sydney, Australia; 4 Department of Health Research Methods, Evidence, and Impact, Faculty of Health Sciences, McMaster University, Hamilton, Ontario Canada; 5 Department of Epidemiology and Data Science, Amsterdam Universities Medical Center, Amsterdam, The Netherlands; 6 Department of Philosophy, Vrije Universiteit Amsterdam, Amsterdam, The Netherlands; 7 School of Medicine, Toronto Metropolitan University, Toronto, Ontario Canada; 8 Institute of Health Policy, Management & Evaluation, Dalla Lana School of Public Health, University of Toronto, Toronto, Ontario Canada; 9 Department of Medicine, McMaster University, Hamilton, Ontario Canada; 10 JAMA and the JAMA Network, Chicago, Illinois USA; 11 Consultant, Lyon, France; 12 Scientific Committee, Institute of Research and Action On Fraud and Plagiarism in Academia (IRAFPA), Geneva, Switzerland; 13 Department of Research in Biomedicine and Health and Center for Evidence-Based Medicine, University of Split School of Medicine, Split, Croatia; 14 School of Epidemiology and Public Health, Faculty of Medicine, University of Ottawa, Ottawa, Ontario Canada

**Keywords:** Artificial intelligence, Attitudes, Chatbots, Peer review, Reviewers, Survey, Perceptions

## Abstract

**Background:**

Artificial intelligence chatbots (AICs) are increasingly being considered for use in scholarly peer review to assist with tasks such as identifying methodological issues, verifying references, and improving language clarity. However, concerns remain regarding their reliability, ethical implications, and transparency. Evidence on how medical journal peer reviewers perceive the role and impact of AICs is limited. This study explored reviewers’ familiarity with AICs, perceived benefits and challenges, ethical concerns, and anticipated future roles in peer review.

**Methods:**

We conducted a cross-sectional online survey of medical journal peer reviewers. Corresponding author information was extracted from MEDLINE-indexed articles added to PubMed within a six-week period. A total of 72,851 authors were invited via email to participate; those who self-identified as peer reviewers were eligible. The 29-item survey assessed familiarity with AICs and perceptions of their benefits and limitations in peer review. The survey was administered via SurveyMonkey from April 28 to June 16, 2025, with two reminder emails sent during the data collection period.

**Results:**

Of 66,679 email invitations, a total of 1260 respondents provided survey responses (response rate: 1.9%), of whom 1157 met the eligibility criteria, and 1077 answered the main survey questions. Most participants were familiar with AICs (86.2%) and had used them for general purposes (87.7%), though most report not using AICs for peer review purposes (70.3%). Most respondents reported that their institutions do not provide training on AIC use in peer review (69.5%), although many expressed interest in such training (60.7%). Perceptions of AIC benefits were mixed, while concerns were widely shared, particularly regarding potential algorithmic bias (80.3%) and issues related to trust and user acceptance (73.3%).

**Conclusions:**

While familiarity with AICs is high among medical journal peer reviewers, their reported use in peer review remains limited. There is clear interest in training and guidance, however, concerns related to ethics, data privacy, and research integrity persist and should be addressed before broader implementation.

## Background

Artificial intelligence (AI) broadly refers to the capability of computer systems or computer-controlled robots to perform tasks typically associated with human intelligence, such as reasoning, problem-solving, generalizing, and learning from past experiences [1, 2]. While current AI programs lack the versatility of human intelligence, specialized applications have permeated numerous fields, including self-driving cars, speech transcription, healthcare, and education [2].

In many domains, AI has demonstrated benefits such as increased productivity, reduced errors, and cost savings. For example, in the field of medicine, AI systems can improve diagnostic accuracy, optimize treatment plans, and reduce healthcare costs for many healthcare systems [3, 4]. These realized and hypothetical benefits have sparked interest in applying AI to scholarly publishing, including the peer review process. Peer review in the context of medical scholarly publishing refers to a quality control protocol whereby independent methodology and content experts are entrusted with reviewing and appraising submitted manuscripts [5]. The “peers” provide arms-length assessments and constructive feedback to uphold the validity and integrity of manuscript content prior to potential dissemination, in accordance with the varying responsibilities and requirements set by the academic publisher or individual journal [5]. Although this remains a promising measure, improvements in both the efficiency and the quality of peer review are very much needed [5].

Artificial intelligence chatbots (AICs), a subset of AI programs, are generative AI tools designed to simulate human conversation via text or speech [6]. AICs have demonstrated versatility in applications ranging from customer service to education [6, 7]. Within scholarly publishing, AICs hold promise for tasks such as improving language clarity, identifying methodological flaws, verifying references, and potentially standardizing review quality [8]. Their integration into peer review workflows could potentially alleviate reviewer workloads, streamline processes, and address longstanding issues such as bias and inconsistency between review reports [8]. Moreover, AICs may address reviewer fatigue and help peer reviewers navigate the increasing volume of requests (e.g., manuscript submissions), ensuring more timely and consistent evaluations [9, 10].

Despite these potential advantages, major concerns include the reliability of AICs in evaluating complex scientific content, the risk of overlooking critical nuances, the potential for manipulation within the peer review process, and ethical considerations regarding their use [11]. For example, well-documented limitations of ChatGPT include generating plausible though incorrect or misleading content (i.e., ‘hallucinating’), citation inaccuracies, and referencing non-existent sources [11–13]. Additionally, as AICs rely on training datasets that may lack novelty or inclusivity, there is a risk of perpetuating outdated information and biases [14].

Ethical concerns also extend to issues of plagiarism, confidentiality, intellectual property rights, transparency, research integrity, and accountability [8]. While the use of AICs in peer review may be acceptable under open peer review models that require authors to consent to the broader sharing of manuscript content, this practice raises ethical and procedural concerns for the peer review of manuscripts received by journals that operate closed (i.e., confidential) peer review models [15]. In such cases, sharing unpublished manuscripts with AICs, particularly those hosted on proprietary platforms that store user input, could compromise reviewer confidentiality and violate copyright and other journal policies [15]. Furthermore, for certain medical articles, reviewers may have access to sensitive patient information that is not intended for publication. Sharing this information with AI systems may constitute a breach of patient privacy and in some jurisdictions, such as the United States, could potentially violate the Health Insurance Portability and Accountability Act (HIPAA) [16, 17].

The scholarly publishing community has started to address these issues through policies for editors and peer reviewers [18–22]. Academic publishers, such as *Nature, JAMA Network,* and *PLOS*, have incorporated guidelines on the use of AICs in their editorial processes, requesting that peer reviewers refrain from uploading any manuscript information to AICs, and that any use of AI for peer review must be disclosed in review forms [18–20]. Other organizations, including the World Association of Medical Editors (WAME) and the International Committee of Medical Journal Editors (ICMJE), have recommended that the use of AICs be prohibited in cases where confidentiality cannot be guaranteed; if reviewers do use AICs, they must have permission from the journal with clear disclosure of how they were used [23, 24]. However, codifying and regulating the responsible use, or the lack thereof, by peer reviewers may become increasingly difficult with the rapidly evolving possibilities and popularity of AICs.

Despite growing interest in the role of AICs in peer review, there remains a scarcity of available research on the attitudes and perceptions of peer reviewers toward their use [25]. As interest in using AI within the domain of scholarly publishing continues to rise, it is crucial to understand how peer reviewers perceive and engage with AICs, along with the impacts they foresee on the peer review process and publishing overall. This understanding will help leverage the potential of AICs while mitigating its limitations, ultimately helping to enhance the quality, transparency, and fairness of the peer review process.

Our study attempted to address this gap by conducting an international cross-sectional survey to assess medical journal peer reviewers’ perceptions of AIC use in peer review processes. The study specifically evaluates ‘AICs’, rather than large language models (LLMs) or generative AI tools, as this refers to conversational, memory-based user interface tools that peer reviewers may interact with using open-ended prompts than underlying LLMs. The primary objective of our study was to assess the current stance of the peer reviewer community surrounding the role of AICs in the medical journal peer review process. To understand peer reviewer attitudes toward AIC use, the framework of our survey incorporated factors such as participant ‘familiarity’ or ‘experience’ with using AICs, their perceived ‘benefits and challenges’, ‘ethical concerns’, and anticipated ‘applications’ for AICs in the peer review process. By providing insights into reviewer perspectives, this study aims to inform the development of ethical guidelines, practical recommendations, and evaluations needed for the possible effective integration of AICs in peer review.

## Methods

### Open science statement

A complete study protocol, with the data analysis plan has been registered on the Open Science Framework (OSF) (https://doi.org/10.17605/OSF.IO/FHC2M) prior to participant recruitment. All relevant study materials and deidentified data can also be found on OSF (https://doi.org/10.17605/OSF.IO/VARJE).

### Ethical considerations

Prior to conducting this study, ethics approval was obtained from the University Hospital Tübingen Research Ethics Board (REB) (REB Number: 080/2025BO2). Informed consent was obtained from all respondents prior to their participation in the survey, as detailed in the email invitation. Participation in the survey was voluntary, and participants had the right to withdraw from the study at any point prior to submitting their completed survey. All data were confidential and anonymous, and no identifying information of respondents was collected.

### Study design

We conducted a large-scale, online, closed, cross-sectional survey of medical journal peer reviewers, sourced from corresponding authors of articles published in MEDLINE-indexed journals who self-identified as peer reviewers, to investigate their attitudes and perceptions toward the use of AICs in the peer review process.

### Sampling framework

A comprehensive list of all journals indexed in MEDLINE (approximately 5300 as of November 2024) was gathered along with their NLM IDs [26]. A search strategy, developed by JYN and reviewed by the rest of the team, of the NLM IDs was executed in OVID MEDLINE (Appendix 1, https://osf.io/varje/files/ncajv). The search was limited to records indexed over the six weeks prior to searching (2024/09/01–2024/10/15).

This time frame was selected because researchers who have published within this period were likely to still be actively engaged in research, providing peer review within their fields and available to respond to the survey invitation. Corresponding authors of all types of research articles were considered for inclusion. For articles with two or more corresponding authors, each author was sent an invitation. Duplicate records were removed prior to the recruitment process. PubMed Identifier (PMID) numbers associated with all yielded articles were exported from OVID as a .csv file and inputted into an R script (created based on the easyPubMed package (easyPubMed, 2023)) to capture author names, affiliated institutions, and email addresses from PubMed [27]. A total of 121,995 PMIDs were retrieved and exported into R [27]. Corresponding author name and email address extraction was completed on December 27, 2024. All generated results were combined into a master list and reviewed for potential errors or duplicates before survey administration.

### Eligibility criteria

To be eligible for participation, participants must have self-identified as medical researchers and served as a peer reviewer of research articles submitted to medical journals. Eligible participants had to have completed and submitted at least one peer review report to at least one professional-level medical journal within the past 24 months at the point of invitation. Those who had peer reviewed exclusively for student journals (e.g., high school, undergraduate or graduate journals) were not eligible to participate.

### Participant recruitment

As PMIDs were collected from all MEDLINE-indexed journals, a range of academic disciplines within the field of medicine were included (e.g., public health, bioengineering, medical education). We used convenience sampling to recruit respondents, targeting corresponding authors identified by our sampling strategy. An email was sent to potential participants containing a recruitment message that outlined the purpose of the study and included a link to the survey, using SurveyMonkey, a secure online survey platform (https://www.surveymonkey.com/) [28]. Upon clicking the survey link, participants were directed to a webpage with an informed consent form. Participants had to indicate consent on the form to proceed to the survey. This was a closed survey; only invited participants were able to participate.

After deduplicating the list, a total of 72,851 corresponding authors were sent invitations to participate, in batches of 10,000. If participants did not respond to the initial invitation email sent on April 28, 2025, reminder emails were sent twice during the weeks of May 5, 2025, and May 12, 2025. A 5-week waiting period followed the final email reminder to accommodate remaining interested respondents before the survey was closed permanently. Overall, participants had a total of seven weeks to complete the survey which was closed on June 16, 2025.

No financial compensation was offered to participants, and participation was completely voluntary and anonymous. Participants could skip the questions that they did not wish to answer and could withdraw from the survey at any time by simply closing the browser window. Participation was anonymous and confidential throughout the study; however, settings allowed for tracking of responded participants (i.e., submission status based on their individual email invitation) for purposes of follow-up reminders and limiting one response per participant. Withdrawal was not possible after the survey was submitted given that the responses were collected anonymously. Email addresses of all invitees were removed from the mailing system following the 7-week window.

### Survey

The complete survey can be found in Appendix 2 (https://osf.io/varje/files/c748y). The survey was created, distributed, and collected using SurveyMonkey (Symphony Technology Group, San Mateo, California, United States of America, https://www.surveymonkey.com/). The first draft of the survey was developed by JYN following a thorough review of available relevant literature, with additional input from experts in AI and peer review. Our review of the literature helped inform the development of questionnaire items by helping identify factors that may influence the adoption or rejection of AIC use, including the user’s familiarity prior experience with AICs, potential applications, and potential benefits and risks with incorporation in the peer review process. All authors of the study reviewed the survey draft, and the survey was pilot tested with 5 individuals external to the study authors who have served as peer reviewers, nominated by authors (CL and AM), to complete the survey in full and provide confidential, constructive feedback regarding the clarity, relevance, and comprehensiveness of the questions prior to circulation. The invited pilot testers’ responses were excluded from the final survey.

The survey consisted of 29 questions which included both closed-ended questions (e.g., multiple choice, yes/no) and open-ended questions (e.g., free-text responses), addressing the following topics:

- Demographic information: Age, sex, country of employment, level of education, primary area of expertise, publication record, and years of experience as a peer reviewer.
- Experience with AICs: Participants’ familiarity with AICs, prior use of AICs in their professional or academic work, and likelihood of permitted AIC use in the peer review process in the future.
- Role of AICs in peer review: Perceptions regarding the potential roles of AICs in peer review, such as aiding in identifying methodological flaws, detecting plagiarism, verifying references, translating research materials, or assessing the quality of writing.
- Perceived benefits of AICs in peer review: Participants’ views on potential benefits, such as reducing workload, improving efficiency, standardizing review quality, addressing biases, and increasing consistency in decision-making.
- Perceived challenges of AICs in peer review: Concerns about the reliability and accuracy of AICs, issues with authorship of peer review reports, risk of amplifying biases, lack of transparency, and potential over-reliance on AI.
- Ethical considerations: Participants’ perceptions of the ethical implications of integrating AICs in peer review, including concerns about accountability, confidentiality, data privacy, intellectual property rights, and their potential impact on the integrity of the peer review process.
- Open-ended questions: Participants had the opportunity to provide additional comments and feedback on the use of AICs in peer review, share their opinions on future integration, and suggest potential areas for improvement or guidelines for ethical use.

Key constructs such as participant familiarity with AICs, and perceived benefits and challenges of AICs in peer review processes were operationalized using Likert-scale response scales (e.g., ‘very familiar to ‘very unfamiliar’ or ‘strongly agree to ‘strongly disagree).

### Data management and analysis

All responses were collected via SurveyMonkey, and the data were exported and analyzed using Microsoft Excel 2007. Descriptive statistics, such as frequencies and percentages, were calculated to summarize the survey response data. Demographic information, such as participant role and years of experience, was analyzed for trends or patterns and described. Descriptive cross-tabulations were conducted to examine any variation in experiences and perceptions related to AIC use by age, sex, career stage and World Health Organization (WHO) region. These cross-tabulations were conducted post-hoc and therefore exploratory. No inferential statistical tests of subgroup differences were conducted. Qualitative data collected through open-ended questions underwent inductive coding and thematic analysis by two authors (DB and ND) [29, 30]. A de-identified version of the survey data can be found in Appendix 3 (https://osf.io/varje/files/wq98x). Personal identifiers provided by participants have been redacted to protect participant privacy. Additionally, crosstabs for key demographic variables (age, career stage, researcher vs. clinician, sex, and WHO regions) can be found in Appendix 4 (https://osf.io/varje/files/c94uk).

To ensure consistency in coding, each author independently coded the responses of the first three survey responses for question 16 and then collaborated to develop a shared codebook based on their results. After developing a consensus on the code(s) and a unified coding framework, all remaining open-ended responses were coded, and the codebook was iteratively updated. Following this, individual codes were grouped into the themes by the two authors independently (DB and ND) and finalized through consensus. Any conflicts were resolved through discussion between the two authors and if needed, by a third author (JYN). The two authors (DB and ND) then developed a clear definition for each theme based on codes incorporated. All data were then reviewed by JYN, DB, and ND. The final manuscript was written and reported in accordance with the Strengthening the Reporting of Observational Studies in Epidemiology (STROBE) and the Checklist for Reporting Results of Internet E-Surveys (CHERRIES) guidelines [31, 32].

## Results

### Respondent demographics

Survey invitations were sent to a total of 72,851 email addresses, of which 1260 invitees provided responses. Of the 72,851 email invitations, 6,172 bounced, leaving 66,679 invitations (response rate: 1.9% or 1260/66,679), and an additional 32,839 emails that remained unopened by the invitee. Hence, only 33,840 email invites were received and opened by potential respondents (response rate: 3.7% or 1260/33,840). Of the 1260 respondents, 1157 met the eligibility criteria (91.8%) to proceed to the remaining 28 survey questions. Of these, 1077 (93.1%) proceeded to answer the main survey questions. Among those that completed all survey questions (844/1077 or 78.4%), the average completion time was approximately 24 minutes. As not all respondents completed all survey questions, the total number of responses for each question varies and is provided in parentheses throughout the presentation of results that follow.

Demographic data are shown in Table 1. The majority of respondents identified as male (*n* = 592 of 1077, 55.0%) and 19 respondents indicated ‘other’ or preferred not to disclose (1.8%). Respondents were employed across the six WHO World Regions, with the greatest representation from the Americas (*n* = 416/1077, 38.6%), followed by Europe (*n* = 377, 35.0%) and South-East Asia (*n* = 111, 10.3%). Approximately half of the respondents stated that English was not their primary language (*n* = 581/1077, 54.0%).

**Table 1:** Respondent demographic data

**Question**	**Answer choices**	**Number**	**Percent**age
Career stage	Early career researcher (< 5 years of formally starting your career post formal education)	199	18.5%
	Mid-career researcher (5–10 years of starting your career post formal education)	257	23.9%
	Senior researcher (> 10 years of starting your career post formal education)	611	56.7%
	Prefer not to disclose	10	0.9%
Total		**1077**	**100%**
Age	< 18	0	0.0%
	18–24	2	0.2%
	25–35	179	16.6%
	36–45	352	32.7%
	46–55	274	25.4%
	56–65	164	15.2%
	65 +	90	8.4%
	Prefer not to disclose	16	1.5%
Total		**1077**	**100%**
Gender	Male	592	55.0%
	Female	466	43.3%
	Other	2	0.2%
	Prefer not to disclose	17	1.6%
	Prefer to self-describe, please specify	0	0.0%
Total		**1077**	**100%**
World Health Organization World Region of primary employment	Africa	38	3.5%
	Americas	416	38.6%
	Eastern Mediterranean	31	2.9%
	Europe	377	35.0%
	South-East Asia	111	10.3%
	Western Pacific	77	7.2%
	Prefer not to disclose	27	2.5%
Total		**1077**	**100%**
Current position	Faculty member at a university/academic institution	739	68.6%
	Academic research staff (e.g., research coordinator) at a university/academic institution)	196	18.2%
	Government researcher	39	3.6%
	Clinician researcher	236	21.9%
	Pharmaceutical industry/company researcher or employee	13	1.2%
	Scholarly communications employee (e.g., scholarly journals/publishing, medical writing)	4	0.4%
	Journal editor or editorial board member	118	11.0%
	Consultant (e.g., own or employed by research consulting firm)	24	2.2%
	Third sector (e.g., NGO, non-profit)	15	1.4%
Total		**1077**	**100%**
Primary research area	Clinical research	559	51.9%
	Preclinical research – in vivo	182	16.9%
	Preclinical research – in vitro	198	18.4%
	Health systems research	140	13.0%
	Health services research	214	19.9%
	Methods research	187	17.4%
	Epidemiological research	257	23.9%
	Other (please specify)	133	12.4%
Total		**1077**	**100%**

Many respondents were senior researchers (*n* = 611/1077, 56.7%), faculty members at a university/academic institution (*n* = 739/1077, 68.6%), that primarily conducted clinical research (*n* = 559/1077, 51.9%), had published more than 50 peer-reviewed research articles (587/1077, 54.5%) and peer-reviewed more than 50 research articles (560/1077, 52.0%). Nearly 1 in 5 (*n* = 199/1077, 18.5%) were also early-career researchers with less than 5 years of formal career experience in scholarly publishing.

### Experience with AICs

Table 2 provides the full details on respondent experiences with AICs and their perspectives on incorporating AICs into the peer review process. Most respondents (*n* = 917/1064, 86.2%) were ‘familiar’ with the concept of AICs, and 25.6% (*n* = 272/1064) stated they were ‘very familiar’ with the concept. Only 2.8% (*n* = 30/1064) were ‘very unfamiliar’ with the concept. The majority had used ChatGPT (OpenAI, California, USA) (*n* = 934/1065, 87.7%) for general purposes. When asked about which AICs they had used for purposes related to peer review, the majority of respondents indicated that they had ‘never’ used an AIC for this purpose (*n* = 744/1058, 70.3%). Of those who had used them for this purpose, 277 responses (26.2%) indicated ChatGPT. Respondents were either ‘likely’ (*n* = 278/1066, 26.1%) or ‘very unlikely’ (*n* = 282/1066, 26.5%) to use AICs for peer review purposes in the future.

**Table 2:** Respondent experience with AI chatbots

Question	Answer choices	Number	Percentage
How familiar are you with the concept of artificial intelligence chatbots?	Very familiar	272	25.6%
	Familiar	645	60.6%
	Unfamiliar	109	10.2%
	Very unfamiliar	30	2.8%
	I’m not sure	8	0.8%
Total		**1064**	**100%**
Which of the following AI chatbots have you used before (for any purpose)? (Please select all that apply)	ChatGPT	934	87.7%
	Google Gemini (formerly Google Bard)	329	30.9%
	Meta AI	137	12.9%
	Microsoft Copilot (formerly Bing Chat)	401	37.7%
	I have never used an AI chatbot	91	8.5%
	Other (please specify)	158	14.8%
Total		**1065**	**100%**
Which of the following AI chatbots have you used before specifically for purposes relating to peer review? (Please select all that apply)	ChatGPT	277	26.2%
	Google Gemini (formerly Google Bard)	37	3.5%
	Meta AI	11	1.0%
	Microsoft Copilot (formerly Bing Chat)	32	3.0%
	I have never used an AI chatbot	744	70.3%
	Other (please specify)	41	3.9%
Total		**1058**	**100%**
How likely are you to use an AI chatbot for peer review in the future?	Very likely	144	13.5%
	Likely	282	26.5%
	Unlikely	209	19.6%
	Very unlikely	278	26.1%
	I am not sure	153	14.4%
Total		**1066**	**100%**
Does your research institution provide any training on how to appropriately use AI tools in the peer review process?	Yes, and I have taken it	60	5.6%
	Yes, but I have not taken it	79	7.4%
	No	741	69.5%
	I am not sure	187	17.5%
Total		**1067**	**100%**
Has your research institution implemented any policies surrounding the use of AI chatbots in the peer review process?	Yes	76	7.1%
	No	611	57.3%
	I am not sure	379	35.6%
Total		**1066**	**100%**

Most respondents reported that their research institution did not provide any training on using AICs appropriately in the peer review process (*n* = 741/1067, 69.5%). Some (139/1067,13.0%) reported that their institution did provide this training; however, only 60/139 (43.2%) had taken this training. Over half of the respondents indicated their research institution had not implemented any policies surrounding the use of AICs in the peer review process (*n* = 611/1036, 57.3%), while only few (76/1066, 7.1%) said that their institution did implement these types of policies.

### Role of AICs in different steps of the peer review process

**Fig. 1:**
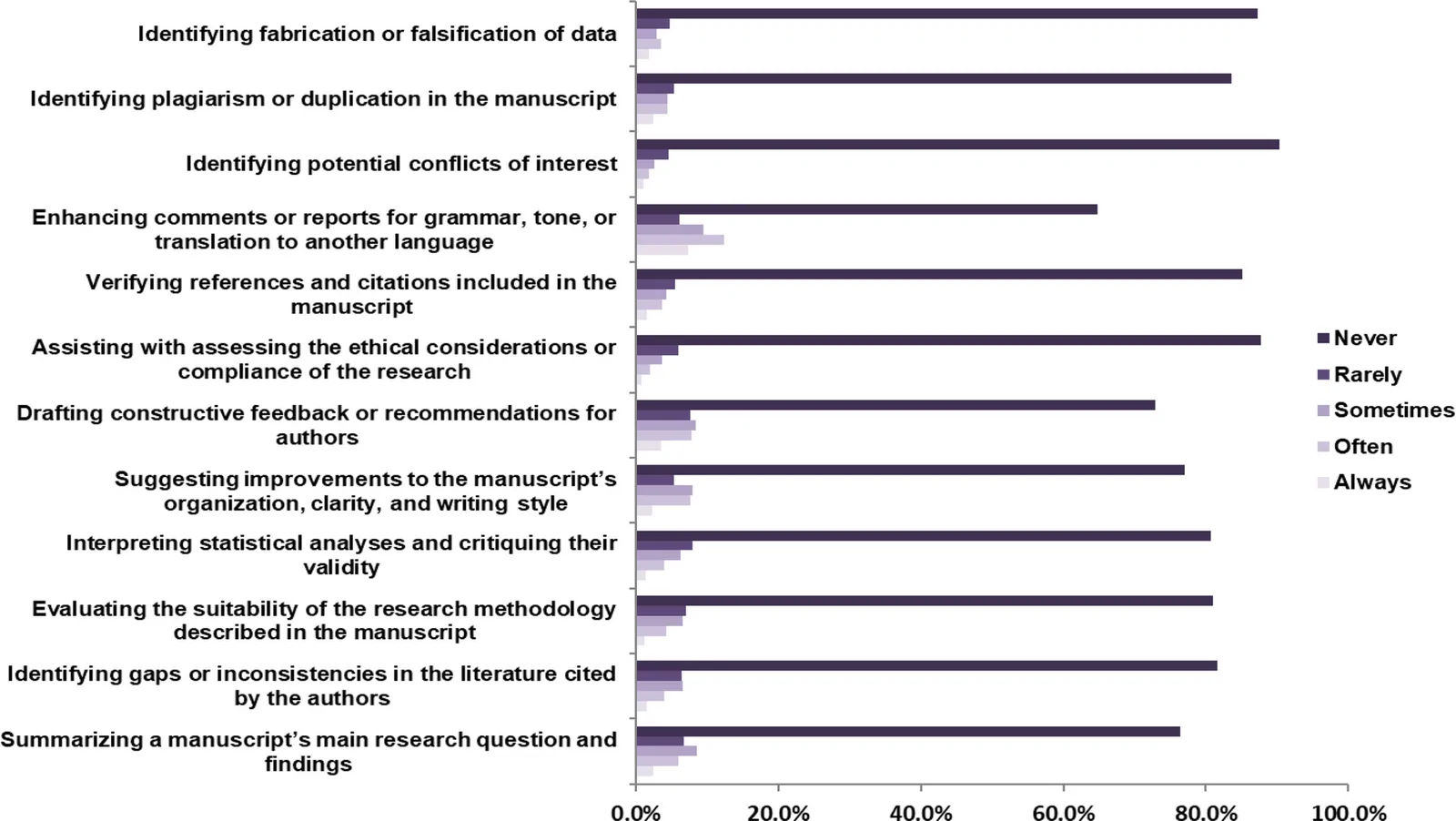
Respondent use of AI chatbots in different steps of the peer review process

All responses regarding how they perceive the role of AICs in the peer review process are shown in Table 3. The respondents were shown a list of 12 different steps of the peer review process and asked to indicate how often they have used AICs to assist them with each step, on a 5-point scale ranging from “Always” to “Never” (Fig. 1). Across all steps, the majority of the respondents stated that they had ‘never’ used AICs to assist them in peer review. Notable findings include that 90.3% (*n* = 933/1033) stated that they had ‘never’ used AICs to assist them with identifying potential conflicts of interest, while 1.0% (*n* = 10/1033) indicated that they ‘always’ used AICs to support them for this purpose. Of the respondents, 87/1039 (8.4%) had ‘always’ or ‘often’ used AICs to summarize the main research question and findings of a manuscript, 158 (15.2%) had ‘sometimes’ or ‘rarely’ used AICs for this purpose, and 794 (76.4%) had ‘never’ used them for this purpose. Regarding providing feedback to the authors, 755/1036 (72.9%) indicated that they had ‘never’ used AICs for this purpose, 165 (15.9%) had ‘sometimes’ or ‘rarely’ used them, and 116 (11.2%) had ‘often’ or ‘always’ used them.

**Table 3:** Role of AI chatbots in the peer review process

Question	Answer choices	Number	Percentage
How much training and education do you think researchers need in order to effectively use AI chatbots in the peer review process?	A lot	396	38.3%
	Some	492	47.5%
	Very little	63	6.1%
	None	23	2.2%
	I am not sure	61	5.9%
	**Total**	**1035**	**100%**
Would you be interested in learning more/receiving training about how to use AI chatbots in the peer review process?	Yes	629	60.7%
	No	166	16.0%
	Maybe	241	23.3%
	**Total**	**1036**	**100%**
How important do you think AI chatbots will be in the future of peer review?	Very important	311	30.1%
	Important	441	42.6%
	Unimportant	72	7.0%
	Very unimportant	43	4.2%
	I’m not sure	168	16.2%
	**Total**	**1035**	**100%**
In general, how do you perceive the potential impact of AI chatbots on the future of peer review?	Very positively	139	13.4%
	Positively	419	40.4%
	Negatively	185	17.9%
	Very negatively	100	9.7%
	I am not sure	193	18.6%
	**Total**	**1036**	**100%**

When asked about how much training and education a researcher would need to effectively use AICs for peer review, 492/1035 respondents (47.5%) answered that ‘some’ training is necessary, while 396 (38.3%) said that ‘a lot’ of training is necessary. Most respondents expressed that they would be interested in learning more/receiving training on using AICs in the peer review process (*n* = 629/1036, 60.7%). Most respondents felt that AICs would be ‘very important’ (*n* = 311/1035, 30.1%) or ‘important’ (*n* = 441, 42.6%) in the future of peer review.

Many respondents reported that AICs would have a ‘very positive’ or ‘positive’ outcome (*n* = 558/1036, 53.9%) on the future of peer review, while 285 (27.5%) indicated AICs would have a ‘negative’ or ‘very negative’ impact, and 193 (18.6%) were ‘not sure’ how AICs would impact future peer review.

### Perceived benefits and challenges of AI chatbots in the peer review process

**Fig. 2:**
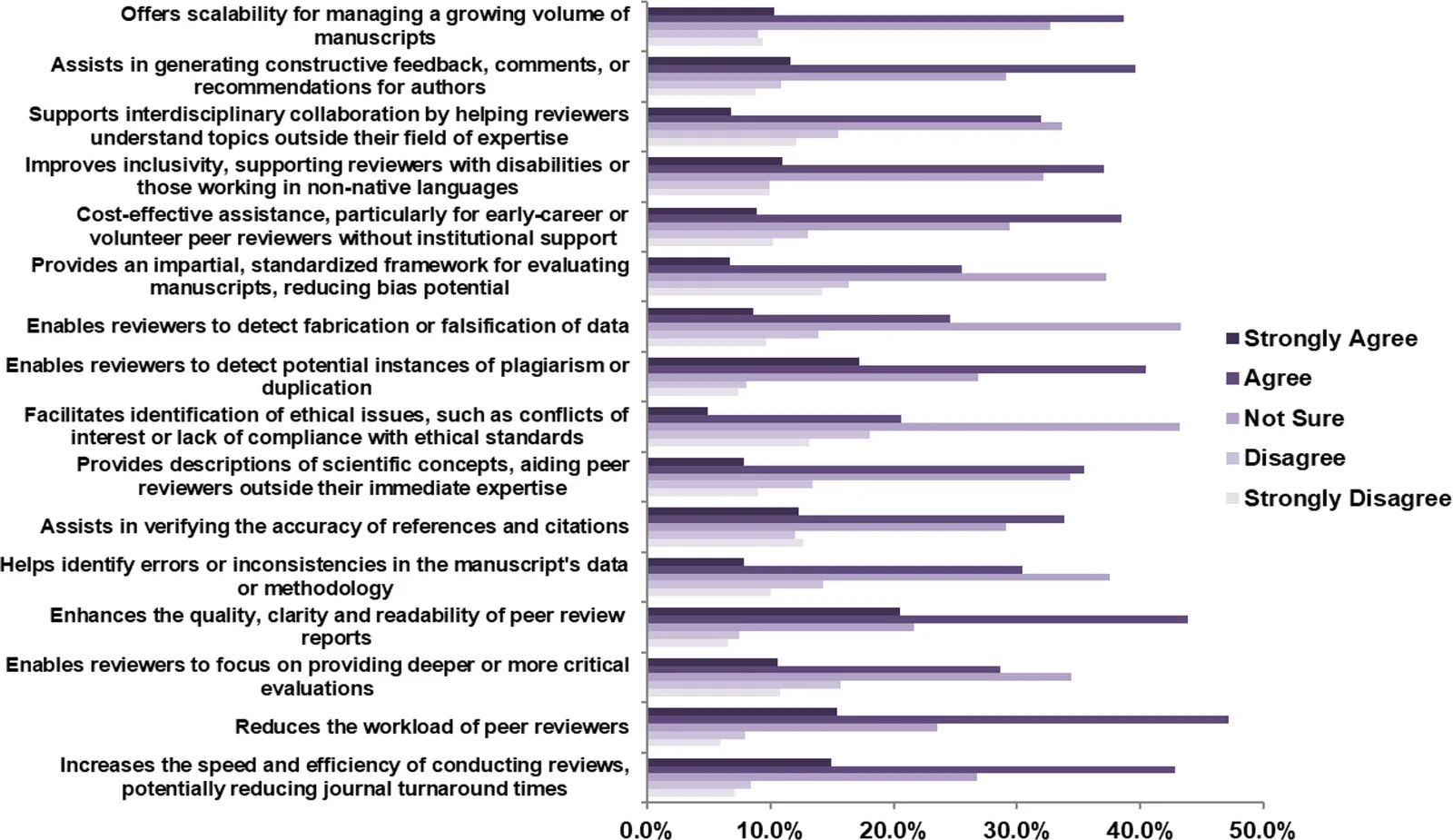
Respondent agreement with potential benefits of using AI chatbots in the peer review process

Respondents rated their agreement with statements relating to how beneficial AICs could be in different steps of the peer review, on a 5-point scale ranging from “Strongly disagree” to “Strongly agree” (Fig. 2). The most common benefits of AICs included enhanced quality, clarity and readability of peer review reports by suggesting edits to grammar, language, tone, or structure (*n* = 652/1013, 64.4%); reducing workload for peer reviewers (*n* = 634/1014, 62.5%); increasing speed and efficiency of conducting reviews (*n* = 586/1014, 57.8%); enabling reviewers to detect potential instances of plagiarism or duplication (*n* = 583/1010, 57.7%); and generating constructive feedback, comments, or recommendations for authors (*n* = 518/1011, 51.2%).

There was a mixed response about whether AICs could allow reviewers to focus on providing deeper or more critical evaluations. Approximately a quarter (268/1014, 26.4%) of respondents disagreed with the statement, while 397 (39.2%) responses agreed, and 349 (34.4%) were ‘not sure’. Also, 379/1011 (37.5%) were unsure if AICs could help identify errors or inconsistencies in the data or methodology of a manuscript, while 387 (38.3%) agreed with the statement, and 245 (24.2%) disagreed with the statement. Respondent views were mixed on whether AICs could assist with the detection of fabrication or falsification of data. Respondent views were also mixed on whether AICs could provide an impartial and standardized framework for evaluating manuscripts and reducing the potential for reviewer bias as 143/1010 (14.2%) responded ‘strongly disagree’, 165 (16.3%) ‘disagree’, 376 (37.2%) were unsure, 258 (25.5%) responded ‘agree’ and 68 (6.7%) ‘strongly agree’.

**Fig. 3:**
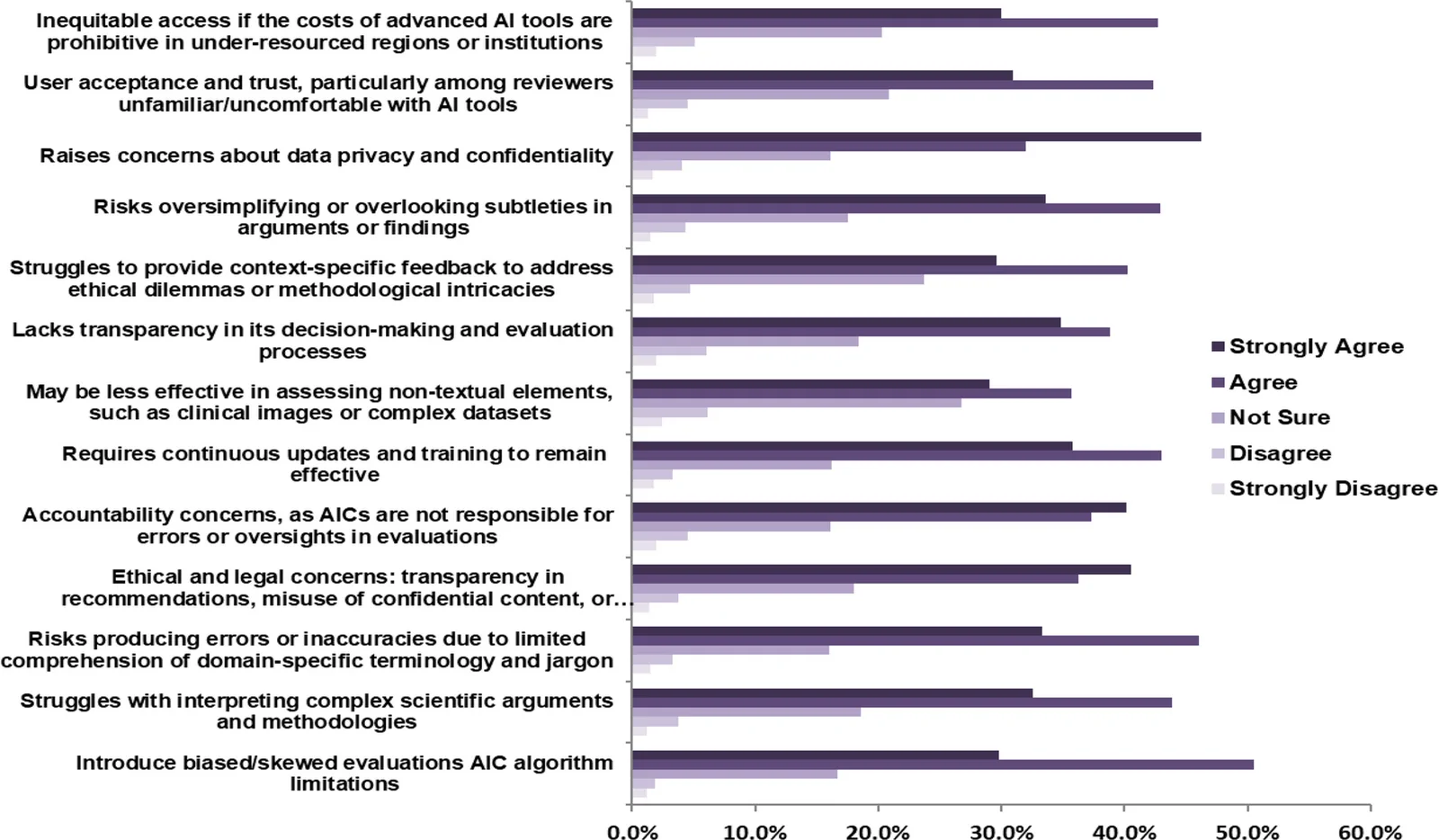
Respondent agreement with potential challenges of using AI chatbots in the peer review process

The respondents rated their agreement with statements relating to potential challenges of using AICs in the peer review process on a 5-point scale ranging from “strongly disagree” to “strongly agree” (Fig. 3). The most agreed upon challenges of AICs included biased or skewed evaluations due to AIC algorithm limitations (*n* = 794/989, 80.3%), risks of producing errors or inaccuracies due to limited comprehension of jargon and limitations inherent to the AICs (*n* = 784/989, 79.3%), requiring continuous updates and training to remain effective with advancements in scientific methods and emerging topics (*n* = 777/986, 78.8%), struggles with the complexity and nuance of interpreting scientific arguments and methodologies in manuscripts (*n* = 756/989, 76.4%), and a risk in oversimplifying or overlooking subtleties in arguments or findings (*n* = 754/985, 76.5%).

### Subgroup analyses

Descriptive cross-tabulations indicated some variation in experiences and perceptions related to AIC use across demographic characteristics (Appendix 4: https://osf.io/varje/files/c94uk). Prior use of AICs for peer review varied by sex, career stage and WHO region. The survey finds that a higher proportion of male respondents (217/592, 36.7.3%) had previously used an AIC for peer review, compared with 114/466 (24.5%) of female respondents. Prior use of AIC for peer review was also more common among early-career researchers (*n* = 73/199, 36.6%) and mid-career researchers (*n* = 101/257, 39.3%) compared to senior researchers (*n* = 155/611, 25.4%). Across WHO regions, prior AIC use ranged from 98/416 (23.5%) in the Americas to 14/31 (45.2%) in the Eastern Mediterranean.

The proportion of respondents who reported being ‘likely’ or ‘very likely’ to use AICs for peer review in the future was also higher among male respondents (*n* = 259/592, 43.8%) than female respondents (*n* = 161/466, 34.5%). This likelihood also similarly varied across WHO regions, ranging from 135/416 (32.5%) in the Americas to 17/31 (54.8%) in Eastern Mediterranean countries. Interest in learning more about and receiving training on AICs in peer review ranged from 80.6% in Eastern Mediterranean (*n* = 25/31), 73.7% in Africa (*n* = 28/38) and 48.3% in the Americas (*n* = 201/416). Differences by career stage were smaller, with similar proportions of early-career (119/199, 59.8%), mid-career (163/257, 63.4%), and senior-career researchers (343/611, 56.1%) indicating interest. Most other respondent perceptions did not differ substantially across demographic characteristics (Appendix 4: https://osf.io/varje/files/c94uk).

### Open-ended questions

The six open-ended questions related to training, policies, use, benefits, and challenges, allowed participants to provide additional feedback and comments on the use of AICs in peer review. Across all questions and responses, 25 themes and 83 codes were identified. Each response was annotated with only one code; each code was grouped under only one designated theme. The timing of each open-ended response was recorded; however, there were no discernible differences in thematic patterns between early and late responses.

Recurring themes included appreciation of AIC roles in peer review processes for ‘proofreading’, ‘learning [manuscript] topics’, ‘content verification’, and additional publisher tools such as ‘translation’ and ‘statistical analysis’ as many believe AICs are ‘increasing opportunities to publish and review diverse research’. However, peer reviewers also shared common concerns: ‘hallucinations’, ‘overautomation of peer review processes’, and ‘compromised research quality assurance’. AICs may also lack the ability to provide context-specific feedback of novel, complex manuscript content as respondents state that “feedback from chatbots can be too general” and “present harmful critique of transformative new ideas”. Altogether, responses were about evenly divided in positive and negative sentiments of AIC use in peer review processes. Broad perspectives included either a ‘favourable view of AI adoption’ (e.g., “helps a lot getting an overview of the work”, “a very good first approach”) and ‘acceptance with caution’, or firm opposition with many echoing that “using [AICs] for peer review is not an appropriate shortcut”, emphasizing the aforementioned concerns and others such as ‘confidentiality’ concerns (e.g., “tantamount to theft…copyrights may not be protected”) and “lack of training [being] a big hurdle” that threaten the integrity of scholarly publishing in medical journals. The themes and code frequencies can be found in Appendix 5 (https://osf.io/varje/files/cehpj). Full results to the thematic analysis tallies can be found in Appendix 6 (https://osf.io/varje/files/u3ypj). A supplemental quantitative analysis of assigned codes for each question can be found in Appendix 7 (https://osf.io/varje/files/23h4y).

## Discussion

This international, cross-sectional survey examined medical journal peer reviewers’ attitudes and perceptions regarding the use of AICs in peer review. Overall, the findings suggest that while peer reviewers are familiar with AICs, uncertainty remains regarding their role in peer review, particularly for tasks requiring in-depth expertise in manuscript evaluation. The findings highlight a need for clearer guidance and training to support the responsible use of AICs.

### Significance and implications of findings

Our survey found that the majority of respondents were familiar with the concept of an AIC and had utilized AICs before for general purposes, with ChatGPT being the most commonly used AIC. However, their limited adoption for uses in peer review suggests significant uncertainty regarding the appropriateness and reliability of AICs. Participants expressed rather polarizing views regarding their future use of AICs in peer review, with most indicating they were either ‘likely’ or ‘highly unlikely’ to use AICs for this purpose, indicating a lack of consensus among the peer reviewer community on whether AICs have a role in supporting peer reviewers without compromising research integrity or the author-reviewer relationship.

In contrast to most comparative literature, the open-ended questions in our survey allowed for further exploration of peer reviewer attitudes and perceptions. For example, the lack of a human factor was a common emerging theme as participants voiced that the evaluation of scientific merit and methodological rigour should remain a human responsibility and should not be offloaded to AICs. Many participants also voiced specific concerns regarding the consequences of peer reviewers, particularly untrained in AIC use, becoming highly dependent on AICs as “an external crutch”, leading to the generation of peer review reports that grossly lack critical thinking required to consider the context of novel, complex research.

Accordingly, respondents had demonstrated greater consensus regarding the limited and supportive role of AICs within peer review processes. Most respondents agreed that the delegation of “low-level tasks”, such as language refinement and improving the readability of reviewer reports are potential acceptable benefits of AICs, provided that content confidentiality and independent reviewer judgement are maintained. However, most also agreed that applications of AICs, such as generating an entire initial reviewer report or uploading novel and complex manuscript content requiring contextual understanding, are higher risk uses that reflect overreliance by the reviewer. This may threaten research integrity, and consequently, erode the trust between authors, peer reviewers, and academic publishers. Moreover, respondents seem to lack consensus on other AIC uses such as offering feedback on reviewer reports or performing a preliminary manuscript assessment for the reviewer, again due to risks surrounding confidentiality, accuracy, and overall reliability of AIC outputs. These may be considered as intermediate-risk applications. Therefore, the current perceptions shared by respondents regarding AIC applications may inform journals, academic publishers, and editorial offices of the primary challenges that must be addressed in developing risk-stratified guidance for peer reviewers regarding permissible uses and expectations surrounding disclosure or accessibility.

Furthermore, most respondents reported that their research institution did not provide training on the appropriate use of AICs in the peer review process. Only 139 respondents reported that their institution provided this training; however, only about half of these respondents had participated in the training. Additionally, very few had reported that their institutions implemented policies surrounding AIC use in peer review. This lack of training and policies surrounding AIC use in peer review, and scholarly publishing in general, could be due to limited awareness or adoption of these tools, especially as many publishing organizations and journals have stated that AI tools cannot be used for research publications [33]. Given that most peer reviewers in our survey expressed that they do not use AICs for peer review, there could be a lack of perceived need for these types of training and policies. Conversely, peer reviewers may refrain from using AICs because of the lack of training and institutional policies, perhaps assuming that AICs are forbidden or discouraged by the journal or academic publisher. Similarly, these resources may be lacking as they may not be considered core academic responsibilities of research institutions and academic publishers, but rather voluntary tasks that must be undertaken by scholars. However, a small number of participants did indicate that they used AICs for peer review, and most participants stated that reviewers did need training and education to effectively use AICs for peer review with most indicating interest in receiving this training. Additionally, the discussion surrounding the use of AICs in scholarly publishing has raised several concerns, including ethical issues, risks of bias, and challenges related to the accuracy and transparency of automated responses, leading many to call for transparency and education regarding their “responsible” use throughout the publishing processes [34].

Recent studies have also raised well-supported concerns regarding the integrity of research involving AIC use due to the lack of action by many academic journals in implementing robust guidelines and controls for AI-generated content [35]. In addition, unreported use and overreliance of AICs has the potential to decrease the quality of peer review, which in turn impacts the credibility of research being published [36]. Although AICs may generate peer reviewer reports following holistic manuscript assessments, there remains concern that AICs may lack the contextual understanding and nuance needed to effectively assess research methodology and framing [35, 36]. The consequences of this deficit may include missed errors or inadequate assessment of key aspects of a manuscript, especially for novel research that requires in-depth, context-specific expertise to provide a thorough evaluation [36].

The quality of the reports generated by the AIC may be a key factor influencing peer reviewers’ perceptions and their willingness to trial and adopt AICs, particularly since the output quality may vary substantially across different AIC models. Therefore, without robust quality control measures implemented for AIC use in peer review and publishing, there remains significant cause for concern regarding the credibility and integrity of research. This limitation highlights the need for quality-control processes to ensure the reliability of AIC-generated reports.

### Comparative literature

Although the potential role of AIC use in the peer review process is a widely discussed topic in the medical scholarly publishing community, there are very few similar studies that have directly explored the attitudes and perceptions of peer reviewers [25, 37–40]. To consider the incorporation of AICs into peer review, it was important to gauge the standpoints of these potential users. Hence, studies incorporating questionnaires, interviews, or surveys were considered comparative literature.

For example, a related study involving a similar international cross-sectional survey of 2165 corresponding authors was conducted in 2023 to understand the attitudes and perceptions of medical researchers toward AIC use in the scholarly publishing process, in general [37]. Most authors perceived a positive potential impact of AICs on the future of scientific research (1236/2048 or 60.4%) and stated that they were likely to use them for upcoming research (1241/2136 or 58.1%). The majority emphasized that training and education would be needed for the effective use of AICs (1713/2049 or 83.7%) but reported that training was not offered by their research institution (1487/2137 or 69.6%) [37]. The study concluded from the survey findings that supportive educational initiatives are warranted for the increasing body of researchers familiar and experienced with AIC use in scientific research.

Similarly, an international survey conducted by the academic publisher *Springer Nature* in March 2025 explored the viewpoints of 5229 researchers on the role of AI tools in the peer review processes of scholarly publishing, non-specific to any discipline [25]. Although approximately 90% of the respondents reported appropriateness of AI use for purposes such as proofreading and translation, with or without disclosure, only 34% of the respondents agreed that it was appropriate to use an AI tool to create an initial peer review report. The majority (52%) stated that using AI to create a peer review report was inappropriate “under any circumstances”, whereas 14% cited inappropriateness “because of privacy concerns” [25]. The majority (57%) also reported that the use of AI tools by peer reviewers to fact-check or clarify questions regarding a specific manuscript (e.g., correct use of a statistical technique) was appropriate. Yet, most respondents reported that they had neither used AI tools nor would consider using them for initial peer review (78%) or to formulate clarifying questions (56%). [25].

Another related international cross-sectional survey, conducted in 2024, exclusively explored the perspectives of 510 Editors-in-Chiefs (EiCs) of biomedical journals regarding the use of AICs in scholarly publishing [38]. Similar to the findings of this survey and other studies [25, 37], the majority of EiCs had not used AICs for editorial purposes (401/479 or 83.7%), though many expressed interest in receiving training (302/469 or 64.4%). Overall, the study reported mixed attitudes as many EiCs would acknowledge benefits of AICs, such as enhancing proofreading abilities, while raising concerns about initial resources required for training, and education and ethical implications of data privacy [38]. Hence, the need for clear guidelines regarding responsible AIC use is again emphasized, in addition to longitudinal assessments of the impact of AIC adoption on the quality and integrity of the editorial or peer review process.

Two additional studies of similar design and purpose surveyed 17 peer reviewers and editors, respectively, of unspecified fields and disciplines, to gauge their general attitudes toward the role of AICs in editing and peer review [39, 40]. Their results were similar as most participants expressed mixed opinions: AICs can enhance the readability and efficiency of peer review reports but lack context-based feedback for authors. Both studies also emphasized that AICs in peer review threaten the essential “human touch” and call for guidelines that ensure responsible use with continued human involvement [39, 40]. Descriptive statistics were not reported.

Finally, the two most recent studies on this topic following the administration of our survey were conducted by academic publishers *Frontiers* and *IOP Publishing* [41, 42]. A global reviewer survey administered by *IOP Publishing* in August 2025 gauged the viewpoints of 348 physical science researchers on the uses of generative AI in peer review processes [41]. Although 32% of respondents admitted to using AI tools for support with peer review tasks, and 40% of respondents perceived a positive impact of AI for peer review, many expressed dissatisfaction regarding the use of AI tools to write (~57%) or augment (~42%) a peer review report for their submitted manuscript [41]. Similarly, an international survey of 1645 active researchers in June 2025 conducted by *Frontiers* found that approximately 53% of reviewers use AI tools in peer review processes, largely by early career researchers (~87%) for synthesizing peer review reports [42]. However, only 21% of respondents agreed that AI use “increases their trust in publishers” with 45% having concerns of misuse [42]. Therefore, both publishers have concluded from their respective studies that although there is an accelerated adoption of AI use by peer reviewers, there remains an urgent need for policy development that can responsibly nurture the potential of AI use for peer review without compromising scientific rigour or research integrity [41, 42].

### Future directions

Concerns remain that the use of AICs may undermine rigorous, unbiased, and transparent peer review practices. Future research should focus on developing ethical frameworks to guide the responsible integration of AICs into peer review, including guidance on their appropriate use, disclosure, and the protection of privacy and confidentiality. Such frameworks could inform journal and publisher policies that balance the potential benefits of AICs with the need to maintain the integrity of the peer review process. Developers should also address the limitations of these tools, including hallucinations and generic or substandard outputs, through ongoing evaluation and improvement. Once these limitations have been sufficiently addressed, repeating a cross-sectional survey using a similar design could help assess changes in peer reviewers’ attitudes and perceptions regarding the role of AICs in peer review. The findings of the present study may also help inform guidance from journals, academic publishers, and institutions on the effective, safe, and responsible use of AICs in peer review. 

### Strengths and limitations

This study used a cross-sectional survey design, which offers several strengths and limitations. One of the key strengths is that this survey approach is cost-effective. As the study only observes a specific population at a single point in time, it was quick to administer and gather data from a large sample of medical journal peer reviewers. As the researchers in our sample are affiliated with diverse medical disciplines and WHO world regions, we received a broad range of opinions regarding the use of AICs in the peer review process, providing valuable insights. Additionally, by collecting the names and email addresses of corresponding authors from publications published within the previous two months only, we minimized the likelihood of encountering inactive or bounced email addresses.

There are also limitations associated with this study. As with all cross-sectional surveys, there is the potential for recall bias and selective non-response bias, which may impact the validity of our findings. Furthermore, self-reported attitudes and perceptions may be weak predictors of implicit biases and actual behaviour. For instance, there may be social desirability bias as peer reviewers may not accurately disclose AIC use for their tasks pertaining to peer review due to their concerns regarding professional and legal consequences, despite the anonymous nature of the survey. Unfortunately, the anonymous nature of the survey also prevented subgroup analyses on open-ended questions that may have provided valuable information on the differing perceptions among certain groups (e.g., early vs senior career researchers). Similarly, although the survey could track response status for reminder purposes, early-versus-late respondent comparison on key closed-ended items was not possible as SurveyMonkey automatically collected the data in the form of descriptive statistics. Another major limitation is that non-English speaking researchers were largely excluded from our sample due to language constraints, which could affect the applicability of our findings to those who primarily publish in languages other than English. Similarly, researchers with limited English proficiency may have experienced difficulties in completing the survey. Our sample strategy involved targeting corresponding authors from a limited publication window, rather than a list of guaranteed peer reviewers, which may introduce coverage error and temporal sampling bias. Moreover, while we made considerable efforts to encourage participation from all invitees, regardless of experience with AICs, the generalizability of our findings may be limited by response bias. Peer reviewers with stronger opinions of AICs, particularly those with major concerns regarding their role in peer review, may have been more inclined to participate, as may active AIC users that are familiar with their potential benefits and applications. In contrast, those with less familiarity or concern with AIC use, uncertainty regarding AIC applications, or hesitancy surrounding their appropriate or permissible use in peer review processes may have been less inclined to participate. Therefore, the proportions of unique, differing perspectives captured by the survey may have been skewed by factors influencing the decisions to respond to our survey, underrepresenting those with lesser engagement, experience, or concern with AICs. The overall response rate was relatively low (3.7%), which further impacts generalizability. However, the final total number of participants nevertheless represents a relatively large sample size due to the scale of our sampling.

## Conclusion

This survey provides insight into peer reviewers’ attitudes and perceptions about AICs for peer review processes in medical journals. Many peer reviewers indicated in this survey that although they had not used AICs for peer review purposes, they were interested in learning more and receiving training on how to use them responsibly to reap the potential benefits in peer review processes. The majority also responded that their research institutes or affiliated journals did not have any training or policies in place regarding the use of AICs for peer review with many expressing concerns surrounding the limitations and ethical downfalls of AIC use medical journal peer review. Hence, given the growing interest, academic journals and publishers may consider developing available training opportunities and policies to help their peer reviewers understand the possible impacts of using AICs in their work of peer review. These steps would be crucial in helping journals responsibly incorporate AICs by preserving the rigour and integrity of peer review processes in medical scholarly publishing.

## Data Availability

All relevant study materials and data associated with this study has been made publicly available on the Open Science Framework, and can be found at https://osf.io/fhc2m.

## Abbreviations

AI: Artificial intelligence
AIC: Artificial intelligence chatbot
ChatGPT: Chat Generative Pre-Trained Transformer
COPE: Committee on Publication Ethics
LLM: Large language model
PMID: PubMed identifier
OSF: Open Science Framework
WAME: World Association of Medical Editors
